# The Spectro-Contextual Encoding and Retrieval Theory of Episodic Memory

**DOI:** 10.3389/fnhum.2014.00075

**Published:** 2014-02-18

**Authors:** Andrew J. Watrous, Arne D. Ekstrom

**Affiliations:** ^1^Center for Neuroscience, University of CaliforniaDavis, CA, USA; ^2^University of Bonn, BonnGermany; ^3^Neuroscience Graduate Group, University of CaliforniaDavis, CA, USA; ^4^Department of Psychology, University of CaliforniaDavis, CA, USA

**Keywords:** hippocampus, oscillations, cell assembly, neocortex, phase-synchronization, cross-frequency coupling, context reinstatement, episodic memory

## Abstract

The spectral fingerprint hypothesis, which posits that different frequencies of oscillations underlie different cognitive operations, provides one account for how interactions between brain regions support perceptual and attentive processes (Siegel etal., 2012). Here, we explore and extend this idea to the domain of human episodic memory encoding and retrieval. Incorporating findings from the synaptic to cognitive levels of organization, we argue that spectrally precise cross-frequency coupling and phase-synchronization promote the formation of hippocampal-neocortical cell assemblies that form the basis for episodic memory. We suggest that both cell assembly firing patterns *as well as* the global pattern of brain oscillatory activity within hippocampal-neocortical networks represents the contents of a particular memory. Drawing upon the ideas of context reinstatement and multiple trace theory, we argue that memory retrieval is driven by internal and/or external factors which recreate these frequency-specific oscillatory patterns which occur during episodic encoding. These ideas are synthesized into a novel model of episodic memory (the spectro-contextual encoding and retrieval theory, or “SCERT”) that provides several testable predictions for future research.

## INTRODUCTION: EPISODIC MEMORY AND CONTEXT REINSTATEMENT

Human episodic memory, the storage, and retrieval of experienced events, is a hallmark of our daily experience. While several factors influence the accuracy of memory retrieval, including the availability of relevant cues, one particularly well-studied factor is similarities between encoding and retrieval. Specifically, memory retrieval is facilitated when it occurs in the original encoding environment (Godden and Baddeley, 1975), an effect called encoding specificity (Tulving and Thomson, 1973). Thus, overlap in sensory and other contextual features between encoding and retrieval is an important determinant of subsequent successful memory retrieval. Yet exactly how this overlap is instantiated within brain neural networks remains unclear.

The hippocampus is a brain region critical to episodic memory (Scoville and Milner, 1957; Vargha-Khadem et al., 1997; Yonelinas et al., 2002) and is thought to underlie this process by binding representations of stimuli with the unique spatiotemporal context underlying an event (Diana et al., 2007; Mitchell and Johnson, 2009; Staresina and Davachi, 2009; Ekstrom et al., 2011). This process is widely assumed to involve interactions between hippocampus and neocortical areas responsible for representing sensory information associated with the original event. An important factor underlying encoding specificity is the reinstatement of similar neural activity to that which occurred during encoding, known as context reinstatement (Marr, 1971; McClelland et al., 1995; Norman and O’Reilly, 2003; Teyler and Rudy, 2007). Thus, we use the term “context” here to refer to both the external environmental factors and internal dynamics which drive neural spiking and oscillatory activity into a specific brain state (Manning et al., 2011). Context reinstatement effects have been well-documented in functional MRI studies (Johnson et al., 2009; Gordon et al., 2013). Moreover, several studies have shown that the same neurons active during encoding are also active during retrieval. For instance, neurons active during encoding of a fear memory were also active during retrieval in rodent hippocampus (Tayler et al., 2012) and memory-related reactivation has also been demonstrated in non-human primates (Hoffman and McNaughton, 2002). In humans, hippocampal neurons that were active during encoding were similarly active during memory retrieval (Cameron et al., 2001; Gelbard-Sagiv et al., 2008). Taken together, these findings suggest that context reinstatement occurs when the same neurons or group of functionally associated neurons (i.e., cell assemblies) that were active during encoding are active again during retrieval. Overall, while the exact overlap of cells that are active during both encoding and retrieval, and how these vary by brain region and even hippocampal subfield, is an area of active research (Tayler et al., 2012), there is little debate that context reinstatement facilitates retrieval and that overlapping neural populations likely underlie this effect.

These core behavioral and neural findings regarding overlap between context and neural patterns of activity between encoding and retrieval, however, leave a fundamental issue unresolved – how are specific cell assemblies formed during encoding selectively reactivated during retrieval via neocortical sensory traces? Two mechanisms utilizing the phase of oscillatory brain activity, cross-frequency coupling and phase synchronization (PS), are hypothesized to underlie these processes. First, the phase of low-frequency activity often modulates the amplitude of high-frequency activity, known as cross-frequency coupling (CFC; also known as phase-amplitude coupling; Bragin et al., 1995; Canolty and Knight, 2010). CFC may promote optimal conditions for synaptic plasticity (Canolty and Knight, 2010; Jutras and Buffalo, 2010) and therefore cell assembly formation. CFC has been observed in numerous regions across several tasks and species (Bragin et al., 1995; Chrobak and Buzsáki, 1998; Canolty et al., 2006; Canolty and Knight, 2010) and is likely to be a general motif in neuronal computation. Second, inter-area PS may support the coordination amongst brain areas (Fries, 2005; Knight, 2007; Womelsdorf et al., 2007; Polanïa et al., 2012; Siegel et al., 2012; Gu et al., 2013) which is likely necessary for hippocampal-neocortical interactions during memory retrieval (McClelland et al., 1995; Buzsáki, 1996; Nadel and Moscovitch, 1997; Eichenbaum, 2000; Nadel et al., 2000; Norman and O’Reilly, 2003). The available evidence thus suggests that CFC and PS represent strong candidate mechanisms for the coordination of cell assemblies during their initial formation and subsequent reactivation.

Emerging evidence indicates that low-frequency oscillatory activity is also reinstated during memory retrieval (Addante et al., 2011; Manning et al., 2011; Morton et al., 2012; Staudigl and Hanslmayr, 2013). Staudigl and Hanslmayr (2013) manipulated the context under which words were encoded and retrieved and showed that oscillations in the theta band (4–8 Hz) at retrieval were differentially enhanced when the encoding and retrieval contexts matched. Whereas theta oscillations during encoding did not index subsequent retrieval success (i.e., hits versus misses), increased theta-gamma cross-frequency coupling (source localized to the left MTL) was also observed for hits compared to misses in both context conditions. Further, gamma power was enhanced at different theta phases between the match and mismatch conditions. These findings and others (Manning et al., 2011; Morton et al., 2012) indicate that low-frequency oscillations may support context reinstatement generally and that anatomically distributed theta-gamma CFC reflects item and content specific processing.

Recent findings have extended these observations, showing that *frequency-specific* patterns of inputs that occur during memory encoding are reinstated during correct retrieval. Wimber et al. (2012) had subjects encode words presented against a 6 or 10Hz flickering background. Successful retrieval of words learned under these conditions recapitulated the frequency-specific patterns of oscillatory phase that occurred during encoding. These findings indicate that one aspect of encoding may rely upon the frequency-specific pattern of inputs that occur during initial stimulus processing, in turn leading to frequency-specific patterns of oscillatory activity. According to context reinstatement, retrieval of these encoded patterns would be maximally effective when similar frequency-specific patterns of activity are reinstantiated. Thus, one mechanism that could guide which ensembles are activated during retrieval, facilitating context reinstatement by reactivating the same neurons that were active during encoding, could be frequency-specific patterns of fluctuations in the local field potential. These findings in turn relate to a recent theoretical proposal, the “spectral fingerprint” hypothesis, which we detail below.

## SPECTRAL FINGERPRINTS IN MEMORY RETRIEVAL

Siegel et al. (2012) have recently suggested that *frequency-specific *oscillatory interactions between the same brain regions may act as a carrier for neural information to mediate different cognitive operations and behaviors. Extending these ideas into the domain of memory retrieval, we have recently provided evidence that human episodic memory retrieval is characterized by frequency-specific interactions between MTL, frontal, and parietal cortices using brain recordings in patients with intractable epilepsy (Watrous et al., 2013a). Patients first performed a spatial navigation task, which provided a novel experience with both spatial and temporal components. During memory retrieval, patients were asked to retrieve either spatial or temporal information, which we have shown to be behaviorally independent in a similar task (Ekstrom et al., 2011; Watrous et al., 2013a). We found robust increases in delta (1–4 Hz) and theta-band (4–8 Hz) phase-synchronization between areas when patients correctly retrieved information from memory. Subsequent findings investigating PS between medial temporal lobe (MTL) and retrosplenial cortex have shown similar results (Foster et al., 2013).

Tallying total functional connectivity for each brain region and adopting a graph theoretic analysis, our results also identified the MTL as a convergence hub for network interactions during memory retrieval. This result is consistent with an influential memory model, Multiple Trace Theory (MTT). MTT argues that the hippocampus is constitutively necessary for memory retrieval by providing the spatial contextual information for a specific memory episode (Nadel and Moscovitch, 1997; Nadel et al., 2000). Further, it argues that the hippocampus binds neocortical feature representations, which are represented by distributed neural ensembles throughout neocortex, into a memory trace. Although MTT has proven highly influential in memory research, its original formulation does not specify the mechanisms by which hippocampal-neocortical binding and interaction occur. Our results, which identified phase-synchronization as a potential coordinating mechanism during retrieval, with the MTL as a hub for these interactions, therefore provide a potential mechanistic basis of MTT. We provide more detail on this idea, and specific predictions generated by incorporating PS into MTT, below.

We also found evidence for spectral fingerprints in memory retrieval. More specifically, retrieving spatial versus temporal information led to frequency-specific changes in low-frequency PS. Spatial retrieval was characterized by delta-band synchronization and temporal retrieval was characterized by theta-band synchronization, raising the possibility of frequency multiplexing in episodic memory. This finding has led us to explore, from a mechanistic perspective, how spectral fingerprints could be implemented in episodic memory encoding and retrieval. Here, we extend the notion of spectral fingerprints in episodic memory by arguing that frequency-specific oscillations not only act as a medium for inter-regional interaction but also represent part of the episodic engram itself.

**FIGURE 1:**
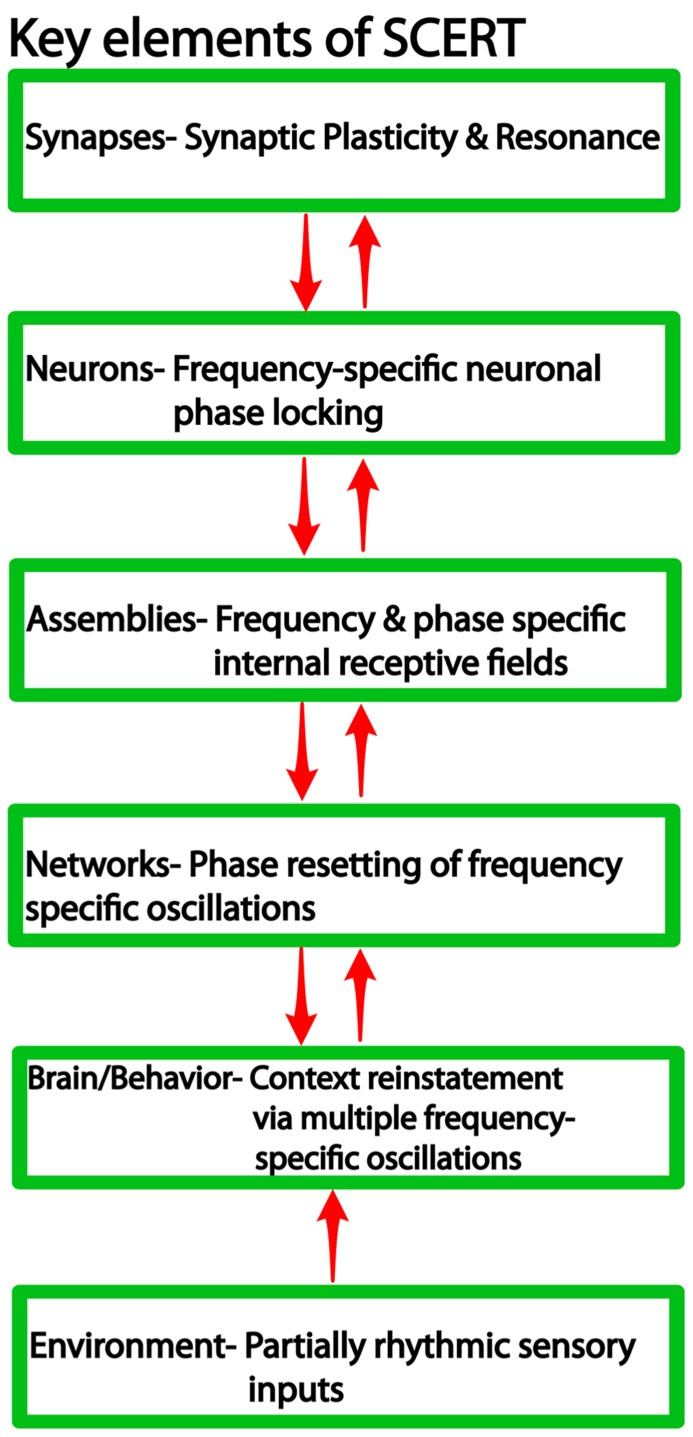
**Levels of organization in neural systems and the associated findings at each level relevant for SCERT**.

We argue that frequency-specificity is a notable feature across several levels of organization (**Figure 1**), including synaptic and neuronal resonance (Hutcheon and Yarom, 2000), frequency and phase specific cell assembly activity (Jacobs et al., 2007; Canolty et al., 2010, 2012), and finally frequency-specific context reinstatement (Wimber et al., 2012) and memory retrieval (Watrous et al., 2013a). Thus, we suggest that context reinstatement is characterized by interactions at multiple levels: frequency coding at the “macro” level to activate the same ensembles that were active during encoding, CFC to activate the correct neurons at the correct phase of the oscillation, and synapse-frequency dependence to activate the correct synapses of the ensemble and thereby reinstate similar patterns of action potential sequences. While definitive answers are forthcoming, we synthesize these ideas into a new framework, the “SCERT,” that provides a number of testable predictions for future research.

## PHASE-CODED REPRESENTATIONS OF ITEMS AND FEATURES

Several studies suggest that the representation of specific items (letters or pictures) or perceptual features results in spatially distributed patterns of high-gamma oscillatory power (Liu et al., 2009; van Gerven et al., 2013) coupled to theta phase (Jacobs and Kahana, 2009). For instance, Jacobs and Kahana (2009) reported that letter-specific gamma activity was phase-locked to theta, arising at the peak of theta and continuing to the theta trough. In another intracranial case study, Jacobs et al. (2012) showed that stimulation at a site in the left temporal lobe elicited memories of the patient’s high school experience. Further, visualization of high school information led to category-specific CFC between theta phase and high-gamma amplitude. These studies indicate that phase-coded information, often found in animal studies, is also relevant for stimulus representation and episodic memory in humans.

There is also evidence that spatially distributed patterns of phase form part of the content of a stimulus representation. Recoding from monkey frontal and parietal cortices during a working memory paradigm, Salazar et al. (2012) have shown that phase-synchronized activity between areas varies as a function of the item held in memory. Moreover, the spatial pattern of oscillatory activity within sensory cortices conveys information about categorical stimulus properties (Ohl et al., 2001) and changes following learning (Freeman and Schneider, 1982). Consistent with these observations, it has recently been proposed that oscillatory activity can act as “metadata” to multiplex a population coded signal representing a stimulus [for review on multiplexing in neural systems, see Akam and Kullman (2014) Nature Reviews Neuroscience]. Collectively, these studies support the hypothesis that frequency-specific oscillatory activity likely carries additional information related to item representations during memory.

Gamma activity in turn appears to be related, at least in some cases, to neuronal firing rate (Crone et al., 1998; Manning et al., 2009; Whittingstall and Logothetis, 2009; Miller, 2010) but see also (Bragin et al., 1995; Henrie and Shapley, 2005; Ekstrom et al., 2009). Thus, spatially distributed low-frequency phase coupled gamma activity, as a manifestation of local neuronal firing, could reflect representations of items or features in the environment. This idea is in turn in agreement with the view that spatially distributed co-activating cell assemblies reflect neural information (Hebb, 1949). Together, these findings implicate phase coding of information via local and distant unit phase-locking, CFC, and phase-synchronization. The exact frequency at which low-frequency phase coding appears to vary based on a number of intrinsic and extrinsic factors (discussed below). As a consequence, initial encoding of information may yield a “spectral fingerprint” of frequency-specific patterns of oscillatory activity that are then reinstated in a frequency-specific manner during retrieval.

## HIPPOCAMPAL EPISODIC ENCODING AND ACTIVE MAINTENANCE

Decades of work have established that the hippocampus is necessary for episodic memory encoding (Scoville and Milner, 1957; Vargha-Khadem et al., 1997; Yonelinas et al., 2002) and recent evidence has indicated hippocampal involvement in “active” working memory maintenance (Ranganath and D’Esposito, 2001; Ranganath and Blumenfeld, 2005; Axmacher et al., 2008, 2010a, 2010b; Cashdollar et al., 2009; van Vugt et al., 2010). Working memory maintenance, which contributes to episodic encoding (Ranganath and D’Esposito, 2001; Ranganath and Blumenfeld, 2005; Ranganath et al., 2005; Axmacher et al., 2008, 2010a, 2010b; Cashdollar et al., 2009; Khader et al., 2010; van Vugt et al., 2010), is associated with theta-gamma cross-frequency coupling and behavioral performance in humans (Axmacher et al., 2010a). Axmacher et al. (2010a) have shown that the frequency of the phase-modulating signal decreases with increasing memory load. The ratio of theta to gamma frequency, however, remained approximately constant, similar to findings in rodents (Bragin et al., 1995) and consistent with models of theta-gamma interactions underlying memory (Jensen and Lisman, 1998). Also consistent with these models, maintenance is associated with periodic replay of maintained items at theta frequency (Jensen and Lisman, 1998; Fuentemilla et al., 2010). Together, these studies are consistent with the idea, discussed above, that individually maintained elements are manifest as gamma oscillatory activity phase-coded by the theta rhythm (Lisman and Idiart, 1995; Lisman and Jensen, 2013). Thus, phase coding of oscillatory activity during working memory maintenance may also be used to promote episodic encoding in the hippocampus and neocortex.

## HIPPOCAMPAL “BINDING” DURING EPISODIC ENCODING

Memory encoding requires the formation of arbitrary associations amongst different types of information. These include items, such as who or what, and contexts, such as when or where. Memory models suggest that the hippocampus may be important for the associational, or “binding,” of these disparate types of information (Diana et al., 2007; Eichenbaum et al., 2007), each of which have been found in the MTL. For instance, single neurons have been found which are selective for different items (Quiroga et al., 2005, 2009; Mormann et al., 2008a, 2011; Gelbard-Sagiv et al., 2008), locations (O’Keefe and Dostrovsky, 1971; Ekstrom et al., 2003; Jacobs et al., 2013), time periods (Macdonald et al., 2011; Kraus et al., 2013), and combinations of these variables (Ekstrom et al., 2003; Macdonald et al., 2011; Kraus et al., 2013). Here, we argue that mechanisms capable of associating the activity of these exemplar classes of neurons, such as CFC and PS, are likely to underlie memory encoding.

### OSCILLATORY MECHANISMS FOR CELL-ASSEMBLY FORMATION

Low-frequency oscillatory activity can dictate the form of synaptic potentiation that is induced, with long term potentiation (LTP) occurring more under conditions of theta activity (Huerta and Lisman, 1993) and when stimulation occurs at the theta peak (Pavlides et al., 1988; Huerta and Lisman, 1993; Huerta and Lisman, 1995). Conversely, synaptic depression is induced at the trough of hippocampal theta (Pavlides et al., 1988; Huerta and Lisman, 1995; Hölscher et al., 1997). Low-frequency phase may also provide a second mechanism for cell assembly formation by promoting spike-timing dependent plasticity (STDP). This form of plasticity relies on coincident spiking between cells with timing in the gamma frequency range (Markram et al., 1997; Caporale and Dan, 2008; Jutras and Buffalo, 2010) and has been indirectly observed in human cortex (Koch et al., 2013). It follows from these studies that mechanisms which coordinate neuronal activity relative to oscillatory phase, such as CFC and PS, influence cell assembly formation (Axmacher et al., 2006; Canolty and Knight, 2010) by regulating synaptic strength and promoting STDP. Together, these studies imply that phase coding, through CFC and phase-synchronization, may be a general mechanism of information processing and memory encoding in the hippocampus (Hasselmo et al., 2002; Harris et al., 2003; Huxter et al., 2003; Lopour et al., 2013).

### PHASE-LOCKED NEURONAL ACTIVITY UNDERLIES MEMORY ENCODING

Several animal studies have found low-frequency phase-locking of single neurons relative to local and distant oscillatory activity relates to visual working memory and/or memory encoding (Jones and Wilson, 2005; Lee et al., 2005; Paz et al., 2008; Siegel et al., 2009; Liebe et al., 2012). Complementary findings have also been observed in human single unit recordings. Rutishauser et al. (2010) have shown that phase-locking of hippocampal neurons to delta and theta (2–10 Hz) oscillatory activity predicts successful encoding of words into human long-term memory. In accord with the hypothesis that working memory maintenance via CFC may also support long-term memory encoding, hippocampal theta-gamma CFC increases during learning of item-context associations in rodents (Tort et al., 2009), suggesting that CFC underlies item-context binding. Similarly, theta-gamma coupling in inferotemporal cortex increases upon learning during a face discrimination task (Kendrick et al., 2011) and theta-gamma power comodulation predicts correct memory retrieval (Shirvalkar et al., 2010). Finally, increases in hippocampal EEG phase-locking (Fell et al., 2008) and hippocampal delta, theta, and gamma-band phase-synchronization (Fell et al., 2001, 2003, 2006, 2008; Jutras et al., 2009) accompanies successful encoding in the MTL.

Additional mechanisms exist for the promotion of specific phase relations amongst neuronal ensembles. Phase entrainment (Lakatos et al., 2008) and/or phase-resetting (Axmacher et al., 2006; Jutras and Buffalo, 2010) of ensemble activity may coordinate specific phase relations between assemblies to ensure that hippocampal inputs arrive at the proper theta phase to promote LTP (Axmacher et al., 2006; Fell and Axmacher, 2011). Consistent with the idea that encoding and retrieval occur on different phases of hippocampal theta (Hasselmo et al., 2002; Manns et al., 2007; Lever et al., 2010; Douchamps et al., 2013), phase resets occur to different phases of theta during memory encoding and retrieval (Rizzuto et al., 2006). Critically, phase resetting has been observed during working memory, encoding, and retrieval (Givens, 1996; Rizzuto et al., 2003, 2006; McCartney et al., 2004; Mormann et al., 2005). In sum, the evidence supports the notion that multiple mechanisms exist to coordinate neuronal activity relative to oscillatory phase during learning and remembering throughout the brain.

## HIPPOCAMPAL-NEOCORTICAL MECHANISMS OF EPISODIC ENCODING

Declarative memory is thought to rely on a distributed hippocampal-neocortical system (Buzsáki, 1996; Eichenbaum, 2000). Thus, it is important to identify mechanisms of cortical encoding and how this relates to hippocampal activity. Recent findings indicate that the local oscillatory environment causally influences cortical neuronal firing, known as ephaptic coupling (Anastassiou et al., 2011). Anastassiou et al. (2011) performed simultaneous intracellular and extracellular recordings from rodent cortical slices *in *vitro while concurrently mimicking an extracellular field via stimulation. The authors found that extracellular fields (particularly below 8 Hz) can phase entrain single neurons and coordinate spiking activity of groups of neurons in the absence of synaptic activity. These results suggest two important inferences. First, ephaptic coupling may provide a mechanism for the hippocampus to coordinate neocortical activity in the absence of structural connectivity, particularly in light of the fact that the hippocampus does not heavily project directly to neocortical areas (Lavenex and Amaral, 2000), which are nonetheless likely to contribute to episodic memory processes. Second, mechanisms that modulate cortical oscillatory activity, such as CFC and PS, may influence the probability of neuronal firing, potentially forming hippocampal-neocortical cell assemblies.

### CROSS-FREQUENCY COUPLING

Theta-gamma coupling is not unique to the hippocampus (Lisman, 2005; Lisman and Jensen, 2013) and recent studies have found increased cortical CFC (Demiralp et al., 2007; Maris et al., 2011; van der Meij et al., 2012) related to novelty (Tsunada et al., 2011) and memory (Canolty et al., 2006; Friese et al., 2013). For instance, Friese et al. (2013) showed that scalp-recorded posterior gamma amplitude is coupled to frontal theta phase during episodic encoding. Moreover, recordings in monkey PFC during working memory showed sequentially maintained items represented as ~3Hz phase-coded spiking and gamma activity (Siegel et al., 2009). These studies illustrate the relevance of neocortical CFC for memory encoding processes.

### PHASE COHERENCE

Phase coherence between areas has been implicated as a mechanism for the hippocampal-neocortical communication thought to underlie learning and memory (Weiss and Rappelsberger, 2000; Weiss et al., 2000; Fell and Axmacher, 2011). Benchenane et al. (2010) assessed rodent learning in a Y-Maze while simultaneously recording from the hippocampus and medial prefrontal cortex (mPFC) of rodents. They found that hippocampal-mPFC coherence increases at the decision point in the maze, particularly following learning, and that mPFC cell pairs showed correlated activity during periods of high-coherence, forming putative coherence-related cell assemblies (CRCA). They further showed that pyramidal neurons shifted their phase-preference to match that of the CRCA. CRCAs were phase-locked to the trough of hippocampal theta, also the phase at which hippocampal cell assemblies operate under some conditions (Harris et al., 2003; Belluscio et al., 2012). Similarly, human cortical activity is also phase-locked to the theta trough (Canolty et al., 2006; Jacobs and Kahana, 2009) and approximately 10% of neurons in monkey prefrontal cortex are phase-locked to hippocampal population firing (Siapas et al., 2005). In summary, similar to its role in coordinating and forming intra-hippocampal assemblies, we conclude that PS provides a coordinating mechanism between previously dissociated cortical and hippocampal cell assemblies during learning (Battaglia et al., 2011).

If memory encoding involves frequency-specific phase coding of neuronal activity, it follows that there should be mechanisms at the cellular and synaptic levels which can modify the frequency preference of neurons. In fact, there is strong evidence to suggest that these mechanisms exist, which provide a critical link between “macro” level oscillations and “micro” level changes at the cell and synapse.

## CELLULAR MECHANISMS OF PHASE-SYNCHRONIZATION AND NEURONAL RESONANCE

At the cellular level, synaptic plasticity is central to learning and memory processes and is partially mediated by NMDA and AMPA receptors (Bliss and Lomo, 1973; Madison et al., 1991; Redondo and Morris, 2011). These receptors have also been shown to mediate phase-synchronization within the hippocampus. Gu et al. (2013) reported *in vitro *evidence that NMDA receptor activation led to delta band phase-synchronization increases between septal and temporal regions of CA3. Similar to previous reports (Sirota et al., 2008; Canolty et al., 2010), synchronization between regions also led to enhanced phase-locking between local units and distal oscillatory phases. These results support the idea that phase-synchronization enhances inter-areal synchronization (Fries, 2005; Womelsdorf et al., 2007) and suggest NMDA receptor mediated PS as a cellular mechanism for intra-hippocampal interactions. As argued above, these interactions may promote synaptic plasticity (Fell and Axmacher, 2011) via LTP or STDP and would therefore also lead to cell assembly formation (Benchenane et al., 2010).

### SYNAPSES AND NEURONS RESONATE AT SPECIFIC AND MODIFIABLE FREQUENCIES

Plasticity leads to short-term facilitation or depression of synaptic weights. Although studied primarily as a mechanism of synaptic plasticity, facilitation, and depression also act as a high and low-pass filter on synaptic activity, respectively, allowing individual synapses to selectively respond to a narrow range of frequency inputs (Markram et al., 1998; Izhikevich et al., 2003), referred to as “synaptic resonance” (Tohidi and Nadim, 2009). Thus, while activity-dependent, short-term plasticity has the effect of strengthening or weakening specific synapses, it also modifies the frequency-preference of these same synapses (Houweling et al., 2002). Individual hippocampal (Chapman and Lacaille, 1999; Pike et al., 2000; Hu et al., 2002; Peters et al., 2005; Wang et al., 2006) and neocortical (Gutfreund et al., 1995; Ulrich, 2002) neurons also display subthreshold resonance in the theta frequency range. Cellular resonance is based upon the passive and active properties of the cell that act as low and high-pass filters, respectively. Factors that influence the active conductance within a cell will therefore affect the cell’s resonant frequency (Hutcheon and Yarom, 2000).

Several channels that contribute to the resonance properties of hippocampal cells, such as NMDA receptors and h-channels mediating the h current (Hutcheon and Yarom, 2000; Wang et al., 2006), are also modified via synaptic plasticity. For instance, inducing LTP in rat CA1 pyramidal neurons led to NMDA receptor-mediated modifications to h channels, resulting in an increased resonance frequency both at the soma and in dendrites (Narayanan and Johnston, 2007). Conversely, induction of long-term depression using 3 Hz synaptic stimulation downregulated I_ h_, significantly lowering the resonance frequency of hippocampal CA1 neurons towards 3 Hz (Brager and Johnston, 2007). These findings were recently extended in a computational modeling study which showed that the frequency preference of model hippocampal neurons increased monotonically with h-channel conductance (Das and Narayanan, 2014). Thus, the frequency preference of hippocampal synapses and neurons are under bi-directional regulatory control by cellular mechanisms also implicated in synaptic plasticity.

### NEURONS THAT RESONATE TOGETHER, OPERATE TOGETHER

These studies have been taken as evidence that hippocampal neurons act as stimulus dependent matched filters which can adaptively alter their frequency response to match the frequency of their inputs (Narayanan and Johnston, 2007). While additional evidence is needed to verify this interpretation, we speculate that the matched filter properties of hippocampal neurons may have interesting consequences for memory encoding. This property of neurons could allow cell assembly formation based on shared frequency preference. In this scheme, rhythmic inputs (detailed below) at frequency *f*_r_ selectively drive a subpopulation of neurons who resonate maximally at frequency *f*_r_. This process would also be expected to increase these neurons’ resonance to *f*_r_ (via LTP and modifications affecting the I_ h_ current) creating a positive feedback loop between resonance and plasticity to promote frequency-specific cell assemblies via Hebbian plasticity. Extending the notion of Hebbian plasticity, we therefore suggest that neurons that resonate together may also operate together in functional cell assemblies.

Consistent with this idea, computational modeling work indicates that network resonance may even develop in networks of neurons which are not intrinsically resonant (Houweling et al., 2002). Moreover, in comparatively simple circuits of resonant neurons, the frequency-preference of a network of neurons tends towards the resonant frequency of individual neurons in the network (Gutfreund et al., 1995; Houweling et al., 2002; Izhikevich et al., 2003; Tohidi and Nadim, 2009). Although the functional consequences of modifying single-neuron and network level resonance have yet to be fully characterized *in vivo*, these studies indicate that frequency-specificity also occurs at the synaptic level of organization in neural circuits over short time scales. Future work employing computational modeling will be necessary to determine how resonance and plasticity interact under conditions of memory encoding and retrieval.

### NEURONAL RESONANCE MODIFIES NEURONAL FUNCTION AND BEHAVIOR

These studies also imply that, rather than simply an epiphenomenon, resonance impacts the functional properties of neurons. In the rodent hippocampus, genetic manipulations that reduce the theta resonance of CA1 neurons also lead to impairments in spatial memory (Peters et al., 2005), demonstrating a strong relationship between neuronal resonance properties and behavior. Other evidence comes from studies investigating entorhinal cortex (EC), the main interface between the hippocampus and cortical areas (Lavenex and Amaral, 2000). The resonant theta frequency, mediated by I_h _(Giocomo and Hasselmo, 2008; Shay et al., 2012), varies along the dorsal-ventral axis of the EC and may regulate the spacing of grid cells (Giocomo et al., 2007). Because these different areas of EC support different aspects of memory and navigation related behavior (Buzsáki and Moser, 2013; Wilson et al., 2013) modifications to the frequency-preference of single neurons would also be expected to impact neuronal computation and behavior. We have argued thus far that frequency-specific processing of inputs is central to episodic encoding. A critical question, then, regards how frequency variability arises in the first place.

## SOURCES OF FREQUENCY VARIABILITY IN NEURAL CIRCUITS

Brain activity can be broadly classified into that which is driven by external, sensory driven input and that which is driven by internal dynamics (Pastalkova et al., 2008; Buzsáki and Moser, 2013). Many aspects of our sensory world, such as natural sounds and speech (Singh and Theunissen, 2003), are inherently rhythmic. Rhythmic stimuli may entrain low-frequency oscillatory activity (Lakatos et al., 2008; Saleh et al., 2010) and CFC. For arrhythmic sensory inputs, rhythmicity may yet be imposed through “active sensing” via motor actions within the environment (Schroeder et al., 2010). For instance, Jutras, Fries, and Buffalo (Jutras et al., 2013) have shown that monkey saccadic eye movements occur in the theta frequency range and lead to hippocampal theta band phase-resetting during memory encoding. Sensory and motor entrainment may therefore impact oscillatory activities in the hippocampus (Ekstrom and Watrous, 2014), in turn setting the stage for enhanced sensory processing and memory encoding.

Seminal studies have shown that various movement-related behaviors elicit different frequencies of oscillatory activity in the hippocampus (Vanderwolf, 1969; Whishaw and Vanderwolf, 1973; Kramis et al., 1975; reviewed in Buzsáki, 2005). Theta frequency increases with increasing movement speed (McFarland et al., 1975; Watrous et al., 2011) and around the time of movement onset and specific behavior-relevant events (Lenck-Santini et al., 2008). Theta frequency may be reduced under novelty (Jeewajee et al., 2008) but see (Penley et al., 2013) and increasing working-memory load (Axmacher et al., 2010a). Functional and/or anatomical factors are also likely to contribute; gamma amplitude is modulated by theta phase in frontal cortex and alpha phase in parietal-occipital cortex (Voytek et al., 2010). Furthermore, the frequency of gamma oscillations dynamically coordinates neurons throughout the MTL (Colgin et al., 2009) and predicts retrieval based upon spatial or sequence memory (Cabral et al., 2014). The frequency of gamma oscillations in different visual cortical areas varies based on attention (Bosman et al., 2012) and stimulus features but is nonetheless dynamically matched between areas (Roberts et al., 2013). By demonstrating frequency locking between cortical areas over fast timescales, the findings of Roberts et al. (2013) establish the viability of matching frequency-specific patterns of oscillatory activity between encoding and retrieval.

Broadly speaking, numerous sensorimotor, anatomical, and behavioral factors (and likely more) potentially contribute to variability in oscillatory frequency patterns. The relative contribution of each factor to frequency variability has yet to be fully explored and warrants further investigation. For our purposes, the key point is that frequency-specific memory-related cell assemblies may arise from numerous intrinsic and extrinsic sources contributing to variability in oscillatory brain activity. We argue that the brain may harness this variability in service of episodic memory encoding such that an engram is also manifest in content specific oscillatory brain states.

## SYNTHESIS

### EPISODIC ENCODING

We have synthesized studies across multiple levels of analysis and species, developing an emerging picture of how the hippocampal-neocortical system encodes and retrieves information. The encoding of a novel experience, such as meeting up with an old friend in a new city, is likely to differentially benefit from hippocampal “binding” of arbitrary associations. The encoding of novel experience, in particular, appears to drive hippocampal activity (Knight, 1996; Ranganath and D’Esposito, 2001), with individual neurons increasing their firing rate to novel stimuli (Rutishauser et al., 2006; Jutras et al., 2009). Environmental novelty alters the phase-preference of rodent CA1 neurons such that they fire closer to the peak of theta (Lever et al., 2010) and successful human memory encoding is associated with 2–10 Hz spike-field coherence at the theta peak (Rutishauser et al., 2010). Novelty may also decrease theta frequency (Jeewajee et al., 2008) which may provide a longer temporal integration window for spiking to occur at the theta peak.

At the assembly level, hippocampal association of items in context is accompanied by theta-gamma CFC (Tort et al., 2009) to induce LTP. Gamma-band PS amongst hippocampal neurons increases in novel spatial environments (Penley et al., 2013) to promote the formation of functional cell assemblies (Axmacher et al., 2006; Jutras and Buffalo, 2010) during successful encoding (Fell et al., 2001; Axmacher et al., 2006; Jutras et al., 2009; Jutras and Buffalo, 2010). Concurrent PS between the hippocampus and neocortex supports the formation of hippocampal-neocortical cell assemblies (Benchenane et al., 2010). Finally, at the synaptic and cellular level, resonance phenomenon contributes to the formation of frequency-specific cellular assemblies such that neurons that resonate together operate together.

### RETRIEVAL OF EPISODIC MEMORIES

The central claim presented here is that frequency-specific patterns of oscillatory activity that occur during encoding are reinstated during memory retrieval. Accordingly, there is evidence for increased CFC (Düzel et al., 2003, 2005; Mormann et al., 2005) and PS (Anderson et al., 2009; Foster et al., 2013; Watrous et al., 2013a) during memory retrieval. The observations of inter-regional neuronal phase-locking and CFC (Sirota et al., 2008) has recently been extended to include more global patterns of oscillatory coupling amongst multiple brain areas (Canolty et al., 2010, 2012). For instance, Canolty et al. (2010) showed that neurons in frontal cortex fire more robustly to specific patterns of global oscillatory phase (termed the neuron’s “internal receptive field,” IRF) recorded simultaneously from widespread cortical areas. The authors found that adjacent neurons, which are putatively embedded in nearly identical local fields, can have very different IRFs, suggesting that global oscillatory patterns may influence single neurons more strongly than local patterns under some circumstances. Firing rates of individual neurons were modulated as a function of frequency-specific patterns of global oscillatory phase. The majority of neurons showed frequency preference for activity <8 Hz, reminiscent of findings demonstrating ephaptic coupling in cortex (Anastassiou et al., 2011). These findings indicate that global, frequency, and phase-specific patterns of oscillatory activity influence single neuron firing which may provide a means for cell assembly activation during memory retrieval.

Several lines of evidence suggest that the hippocampus may orchestrate the selection of specific cell assemblies during retrieval (Nadel and Moscovitch, 1997; Canolty et al., 2010; Battaglia et al., 2011) by promoting frequency-specific oscillatory patterns in neocortical areas. First, the hippocampus is well positioned anatomically in the associational cortical hierarchy (Felleman and Van Essen, 1991; Lavenex and Amaral, 2000). Second, the human hippocampus has been observed to show peak oscillatory activity and to function at several frequencies in the 1–10 Hz band (Jacobs et al., 2007; Axmacher et al., 2010a; Rutishauser et al., 2010; Lega et al., 2011; Watrous et al., 2013b). Third, local delta (Gu et al., 2013) and theta generators with distinct behavioral correlates have been observed in the hippocampus (Mormann et al., 2008b; Goutagny et al., 2009; Montgomery et al., 2009; Watrous et al., 2011). Human intracranial recordings from the neocortex and studies in neocortical slice preparations also indicate that theta is generated mostly locally (Bao and Wu, 2003; Raghavachari et al., 2006) and may occur with multiple peaks in the theta band (Groppe et al., 2013) which could allow independent coordination of multiple cortical regions. While the exact mechanisms of hippocampal to neocortical PS remain unclear, it may be possible for the MTL, via hippocampal projection interneurons, to enhance low-frequency oscillations in neocortical regions by entraining cortical interneurons, in turn driving local cortical activity via ephaptic coupling (Blatow et al., 2003; Tierney et al., 2004). We therefore suggest that the necessary circuitry is in place for intrinsic activity patterns in the hippocampus to drive cortical activity to reinstate cortical oscillatory patterns and select a specific cell assembly.

The demonstration of IRFs and similar findings during motor tasks (Canolty et al., 2010, 2012) provides evidence that single neurons are more active under specific, global oscillatory conditions. This raises the possibility that cortical-hippocampal cell assemblies formed during encoding may be similarly activated or selected during memory retrieval by reinstating the global, frequency-specific oscillatory patterns present during encoding. Consistent with the spectral fingerprint hypothesis, Voytek et al. (2010) showed that high-gamma power is phase-locked to multiple low-frequency rhythms at the same time, a version of frequency multiplexing. These findings suggest that cell assemblies may be selected simultaneously based on their response to specific frequencies and phases (Engel et al., 2001; Canolty et al., 2010; Canolty and Knight, 2010; Voytek et al., 2010; van der Meij et al., 2012; Jirsa and Müller, 2013; Watrous et al., 2013a).

## THE SPECTRO-CONTEXTUAL ENCODING AND RETRIEVAL “SCERT” THEORY

**FIGURE 2:**
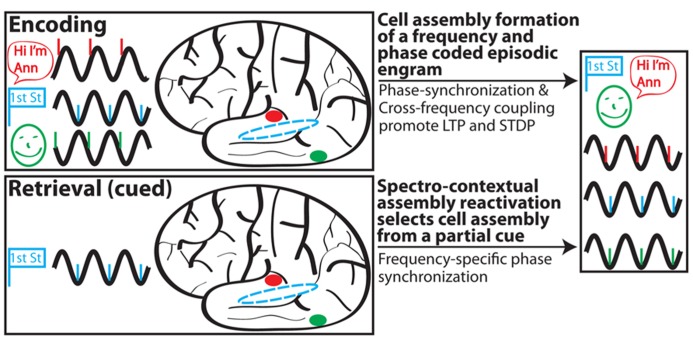
**Schematic depiction of SCERT.** In this scenario, a person perceives (via active sensing) and encodes a woman (green) who introduces herself as Ann (red) on the corner of first street (blue). Associated activity in different brain areas is shown for each type of information in a different color; stimulus representations are shown in prototypical brain areas for clarity but may also be more diffusely distributed throughout the brain. Phase-locking of neuronal ensembles for each region is shown for the initial representation and following learning. Episodic encoding is associated with phase synchronization between areas and CFC between assemblies representing different aspects of the experience. During retrieval, a partial cue (encountering first street) initially drives hippocampal activity to reinstate the spectral signature associated with the encoding experience. Note that some aspects of SCERT, including neuronal resonance and frequency multiplexing, are not depicted.

Drawing upon the ideas of context reinstatement, spectral fingerprints and multiplexing, MTT, and internal receptive fields, we propose the following model (**Figure 2**) of episodic memory:

(1) The rhythmic external world and/or rhythmic sensory sampling (active sensing) impose rhythmicity onto sensory inputs. Phase entrainment and phase resetting serve as mechanisms for rhythmic inputs to arrive during periods of heightened local excitability.
(2) Rhythmic inputs act as a frequency and phase specific spectral context during encoding and selectively drive synapses and neurons with similar resonant properties. Neurons that resonate together will also operate together in functional cell assemblies.
(3) Shared frequency preference amongst neurons, CFC, and PS contribute to the formation of a cellular assembly for a specific engram through Hebbian plasticity and STDP. This encoding process strengthens synaptic connections between neurons in the assembly and also modifies their resonance properties to match that of the assembly.
(4) Reactivation of a cell assembly during retrieval is a hippocampally driven process whereby the hippocampus reinstates, largely based on contextual cues, the internal receptive field for a given cell assembly. This process is frequency multiplexed, allowing for the simultaneous reactivation of different assemblies operating at different preferred frequencies.

Spectro-contextual encoding and retrieval theory makes several predictions for future research which can be tested using both correlative and causal methods. First, providing retrieval cues based on real-time detection of brain states should augment memory performance. For instance, presenting a retrieval cue during a window in which oscillatory phases match those present during item encoding should selectively facilitate memory performance for the item. This prediction should be testable in existing datasets.

Second, SCERT predicts that neuronal ensembles that resonate together also operate together. Although difficult to test at the macroscopic level in humans at present, it is in principle possible to identify several single neurons in humans, identify their frequency and phase preference, and then determine how neurons with similar spectral preferences operate during behavior. Neurons that show multiple frequency and phase-preferences during different behaviors would also provide evidence for frequency multiplexing. Thus, a novel prediction here would be that ensembles representing different components of memory, if stimulated together at the same frequency, could induce novel memories. For example, if one could stimulate representations for “airplane” and “purple stripes” one could create a false memory of a novel purple-striped vehicle even though this had never been experienced before. Although there is evidence to suggest that stimulation can sometimes induce retrieval of remote memories (Jacobs et al., 2012), the veracity of these recalled memories is not typically investigated. Thus, to our knowledge, there is currently no evidence to suggest that DBS can induce recollection of never-before-experienced memories. Based on our theoretical framework, however, we believe that it is reasonable to expect that DBS, perhaps if timed with endogenous hippocampal oscillations using intracranial recordings, could induce veridical memory recall.

There are additional predictions that can be made using causal stimulation methods. It should be possible to manipulate memory performance by experimentally controlling the frequencies under which memories are encoded and retrieved. This could be accomplished either by experimentally controlling the frequency of stimulus presentation or via electrical stimulation methods. For instance, stimulating the hippocampus via deep brain stimulation (DBS) during retrieval at a frequency introduced during encoding should facilitate retrieval by reactivating frequency-specific cortical traces. Thus, if recordings determine that a specific spatial context (say a scene) is encoded via 3–5 Hz synchronization between the hippocampus and specific ensembles within neocortex, stimulating the hippocampus in concert with these cortical nodes should enhance recollection of the scene and any previously encoded details with this scene. No studies to date have matched stimulation and recordings in the human brain and so this prediction remains novel and untested. Yet, it provides an important link between oscillatory coherence, cortical, and hippocampal cooperation.

Conversely, we predict that inducing a mismatch between encoding and retrieval frequencies will impair memory retrieval by producing a spectral brain state that does not selectively drive the assembly at its preferred IRF. For example, if a scene were encoded via 3–5 Hz coherence between hippocampus and cortex, stimulating either hippocampus or cortex at this frequency, provided this simulation is out-of-phase with the on-going hippocampal activity, will impair contextual retrieval. During retrieval, a similar effect would result from stimulating at a high frequency that is inconsistent with the encoded frequency. In fact, studies using DBS in hippocampus suggest that stimulating the hippocampus at fast frequencies will disrupt encoding of pictures, which is possible if stimulation occurs out of phase with on-going cortical stimulation or at a different frequency all together from what would typically be induced in cortex (Coleshill et al., 2004). Thus, a novel prediction of SCERT is that stimulating hippocampus out-of-phase phase with cortex, either during encoding or retrieval, will result in impaired memory retrieval.

Finally, episodic encoding should benefit from exogenously induced phase-synchronization between neocortical areas in patients with hippocampal damage or dysfunctional hippocampi. Specifically, inducing neocortical synchronization via methods such as transcranial magnetic stimulation (TMS) or transcranial alternating or direct current stimulation (tACS/tDCS) may facilitate encoding of neocortical memory traces, although these are likely to be more general and semantic in nature. These methods have been used previously to induce human cortical theta PS and causally modulate performance on a simple memory task (Polanïa et al., 2012). To the extent that the hippocampus normally provides spatiotemporal contextual information and coordinates neocortical assemblies during encoding, frequency-specific entrainment of neocortex in these patients may facilitate memory trace formation for some (non-contextual) aspects of an encoding experience. Consistent with our prior prediction, this memory trace may be reactivated in these patients by artificially driving activity to reinstate the IRF of the neocortical memory trace. Thus, SCERT also provides a new prediction for how to partially remediate episodic memory impairments in clinical populations.

## OUTSTANDING QUESTIONS

What are the broader mechanisms responsible for this assembly selection process? We suggest two possibilities to this yet unresolved question. Environmental cues may reactivate a subset of neurons in the cell assembly encoding a specific memory. For instance, seeing a picture of one’s high school prom date may activate neuronal populations or specific oscillatory patterns that represent the person. If neurons which resonate together also operate together, this process may ultimately drive activity in the larger, frequency-responsive assembly representing the entire prom experience. This speculative possibility warrants further studies. Second, in the absence of closely associated or rhythmic environmental cues, it may be possible for intrinsic brain dynamics or top-down processes to recreate oscillatory patterns from encoding via local generators of theta activity as discussed above. Given that many cues are not inherently rhythmic, some other source of rhythmicity may be at play, possibly top-down signals from associational cortical areas or active sensing processes (Schroeder et al., 2010; Jutras et al., 2013); these possibilities await further investigation.

Our model of memory encoding and retrieval raises a number of intriguing new questions for future research. First, how does sleep-related reactivation fit into our model? Does memory replay reinforce the spectral patterns which were originally present during encoding? Second, what are the constraining conditions for multiplexing in memory (Schyns et al., 2011; Knight and Eichenbaum, 2013)? Third, what roles do modulations in power and phase play in neuronal computations, and more specifically, what accounts for the observed increases and decreases (Sederberg et al., 2003; Lega et al., 2011; Burke et al., 2013) in low-frequency power during memory tasks? Fourth, what do CFC and PS independently contribute to encoding and retrieval? Fifth, given that LTP and resonance appear to be inter-related phenomenon, what are the conditions in which they share diverging or converging roles in promoting assembly formation and reactivation?

Finally, unique patterns of oscillatory phase are almost always present in the brain. This activity may be capable of selectively driving assemblies with a specific internal receptive field (Canolty et al., 2010). Why aren’t memories spontaneously recalled more often? Future studies will be necessary to address this open question but we offer two speculative possibilities. First, because retrieval cues are needed to drive cell assembly activity to begin with, oscillatory phase dynamics in the absence of cues may not be sufficient to drive specific cell assemblies. Another possibility, consistent with MTT, is that cortical cell assemblies are indeed driven by ongoing oscillatory phase but memory retrieval itself, involving the tying together of these details into a specific episode, requires hippocampal involvement.

In summary, SCERT argues that memory encoding is subserved by synaptic and neuronal resonance, CFC, and PS such that frequency-specific oscillatory activity both forms cell assemblies and contributes to the episodic engram. We provide evidence that a range of factors, including synaptic and neuronal resonance, sensory, motor, and behavioral factors, contribute to frequency-specific oscillatory dynamics. Implementing the ideas of context reinstatement, we argue that retrieval occurs via the hippocampally driven reinstatement of an engram’s spectral signature, ultimately driving activity in spatially distributed cell assemblies that code for a specific experience.

## Conflict of Interest Statement

The authors declare that the research was conducted in the absence of any commercial or financial relationships that could be construed as a potential conflict of interest.
